# Yeast oral vaccines against infectious diseases

**DOI:** 10.3389/fmicb.2023.1150412

**Published:** 2023-04-17

**Authors:** Nicanor Austriaco

**Affiliations:** Department of Biological Sciences, UST Laboratories for Vaccine Science, Molecular Biology, and Biotechnology, Research Center for the Natural and Applied Sciences, University of Santo Tomas, Manila, Philippines

**Keywords:** yeast, oral vaccine, *Saccharomyces boulardii*, *Saccharomyces cerevisiae*, vaccine, vaccine adjuvant and delivery system

## Abstract

Vaccines that are delivered orally have several advantages over their counterparts that are administered *via* injection. Despite the advantages of oral delivery, however, approved oral vaccines are currently limited either to diseases that affect the gastrointestinal tract or to pathogens that have a crucial life cycle stage in the gut. Moreover, all of the approved oral vaccines for these diseases involve live-attenuated or inactivated pathogens. This mini-review summarizes the potential and challenges of yeast oral vaccine delivery systems for animal and human infectious diseases. These delivery systems utilize whole yeast recombinant cells that are consumed orally to transport candidate antigens to the immune system of the gut. This review begins with a discussion of the challenges associated with oral administration of vaccines and the distinct benefits offered by whole yeast delivery systems over other delivery systems. It then surveys the emerging yeast oral vaccines that have been developed over the past decade to combat animal and human diseases. In recent years, several candidate vaccines have emerged that can elicit the necessary immune response to provide significant protection against challenge by pathogen. They serve as proof of principle to show that yeast oral vaccines hold much promise.

## Introduction

Vaccines that are delivered orally have several advantages over their counterparts that are administered *via* injection (Wang and Coppel, 2008; Alotaibi et al., 2021). Physiologically, oral delivery of vaccines can stimulate both the humoral and cellular immune response at both systemic and mucosal sites, which, in principle, should establish broader and longer-lasting protection (Russell and Mestecky, 2022). They are also non-invasive, convenient, and can be self-administered. These are characteristics of a vaccine that can help increase immunization coverage of a population during a pandemic: It is striking that a recent study of 15,000+ adults in the United Kingdom revealed that injection fears can explain 10% of COVID-19 vaccine hesitancy (Freeman et al., 2021). These are also characteristics that would benefit developing, i.e., low- and middle-income, countries (LMICs), which often have poor healthcare systems that lack healthcare workers (Levine, 2010).

Despite the advantages of oral delivery, approved oral vaccines are currently limited to diseases that affect the gastrointestinal tract (GIT) or to pathogens that have a crucial life cycle stage in the GIT (Vela Ramirez et al., 2017). Moreover, all of the approved oral vaccines for these disease involve live-attenuated or inactivated pathogens. However, there are a variety of delivery systems that are being developed for oral delivery of vaccines including particle-based, lipid-based, adenoviral-based, and bacterial-based vectors (Coffey et al., 2021). Many of these emerging oral delivery systems utilize subunit vaccines involving components of the pathogen rather than whole-viral or whole-cell formulations.

This mini-review summarizes the potential and challenges of yeast oral vaccine delivery systems for human and animal infectious diseases. These delivery systems utilize whole yeast recombinant cells that are consumed orally to transport candidate antigens to the immune system of the GIT. The review begins with a discussion of the challenges associated with oral administration of vaccines and the distinct benefits offered by whole yeast delivery systems, particularly the commonly used budding yeast, *Saccharomyces cerevisiae*, over other delivery systems that rely on particles, lipid vesicles, viruses, or bacterial cells. It then surveys the emerging oral vaccines that have been developed over the past decade using yeast to counter animal and human diseases. In the past few years, several candidate vaccines have emerged that can elicit the necessary immune response to provide significant protection against challenge by pathogen. However, there is a need for studies involving adjuvants and nutritional supplements to try to optimize the use of yeast oral vaccines, especially if they are to move to human clinical trials and beyond.

## The inherent advantages of yeast oral vaccines

There are four fundamental biochemical and physiological challenges that need to be overcome by any efficacious oral vaccine targeting the small intestine, which is the target region of the mammalian GIT for all currently approved oral vaccines and for all the emerging yeast candidate delivery systems. First, a candidate oral vaccine must survive exposure to low pH, bile salts, and digestive enzymes including peptidases and other proteolytic enzymes, as it traverses the gut. Many candidate immunogens such as antigenic proteins and peptides are highly susceptible to degradation and denaturation in this environment (Renukuntla et al., 2013; Yang et al., 2022). Second, once it reaches the gut, the candidate vaccine has a time limit for absorption that is determined by the relatively brief residence time (3–4 h) in the small intestine (Tyagi et al., 2018; Vinarov et al., 2021). If the immunogen is not absorbed in time, then it cannot be presented to the immune system.

Third, upon absorption, the oral vaccine must deliver the immunogen across the intestinal mucosa, which is structured to prevent the unwanted uptake of pathogens and macromolecules, so that the antigen can interact with the gut-associated lymphoid tissue (GALT) and be presented to the immune system. Finally, an oral vaccine must overcome the complex mechanisms of immune tolerance that allow human beings to live alongside commensal organisms and the benign environmental antigens found in food (Tordesillas and Berin, 2018; Foong and Santos, 2022; Zhao and Maynard, 2022). It is likely that this final challenge explains the higher doses of antigen (sometimes up to 100-fold) required for immune stimulation by oral vaccines as compared to their injected parenteral counterparts (Pavot et al., 2012).

Yeast delivery platforms have at least three inherent advantages over other oral platforms that rely on particle, lipid vesicle, and viral or bacterial systems. We will focus here on the budding yeast, *Saccharomyces cerevisiae*, which is the most popular yeast being developed into oral vaccines at this time (Table 1). Two cousins of this budding yeast, *Pichia pastoris* and *Kluyveromyces lactis*, are also being tested as oral vaccines in a handful of animal models (Seo et al., 2013; Zhao et al., 2014; Wang et al., 2016; Embregts et al., 2019; Ananphongmanee et al., 2021), but much less is known about the interactions of these yeasts with the mammalian digestive and immune systems.

**Table 1:** Candidate yeast oral vaccines for animal and human diseases (2013–2023).

Yeast species	Vaccine design strategy (living or dead yeast cells)	Pathogen	Antigen	Immune response	References
*Saccharomyces cerevisiae*	Surface display (alive)	SARS-CoV2	RBD-FP of the Spike protein (receptor binding domain-fusion peptide)	Upregulated IgG and IgA response	Zhang L. et al. (2022)
	Surface display (dead)	*Eimeria tenella*	EtAma1; EtImp1; EtMic3	None Reported	Soutter et al. (2022)
	Surface display (alive)	Fowl adenovirus (FAdV)	Fiber-2 protein	Upregulated IgG and sIgA response; upregulated IFN-α, IFN-β, and CD8	Cao et al. (2022)
	Surface display (alive)	Cyprinid herpesvirus 2 (CyHV-2)	pORF25	Upregulation of 16 immune- and antivirus-related genes	Dong et al. (2022)
	Surface display (dead)	SARS-CoV2	RBD of the Spike protein (receptor binding domain)	Upregulated IgG and IgA response; upregulated IFN-γ and IL-4	Gao et al. (2021)
	Surface display (alive)	African swine fever virus (ASFV)	P30-Fc𝛾; P54-Fcα fusion proteins	Upregulated IgG and IgA response	Chen et al. (2021)
	Surface display (dead)	White spot syndrome virus (WSSV)	pVP28-pVP24 fusion protein	Upregulated SOD and PO response	Lei et al. (2021a)
	Surface display (dead)	*Theileria parva*	Tp1, Tp2, Tp9, Tp10 and N36	Upregulated IgG response	Goh et al. (2021)
	Surface display (dead)	H5N1 influenza A virus	HA protein	Upregulated IgG and IgA response; upregulated IFN-γ and IL-4	Lei et al. (2021b)
	Surface display (dead)	*Helicobacter pylori*	UreB and VacA	Upregulated IgG and IgA response	Cen et al. (2021)
	Surface display (dead)	White spot syndrome	pVP28	None reported	Le Linh et al. (2021)
	Surface display (dead)	H7N9 influenza A virus	HA protein	Upregulated IgG response; proliferation of IFN-γ/IL-4 cells	Lei et al. (2020)
	Surface display (dead-encapsulated)	Cyprinid herpesvirus 3 (CyHV-3)	pORF65	Upregulated IgG response; Upregulation of 5 immune-related genes	Ma et al. (2020)
	Surface display (alive)	Koi herpesvirus (KHV)	pORF131	Upregulated IgM response	Liu et al. (2020)
	Surface display (alive)	*Toxoplasma gondii*	TSR domain of TgMIC16	Upregulated IgG response; proliferation of CD4^+^ and CD8^+^ cells	Wang et al. (2018)
	Surface display (alive)	Dengue virus (DENV)	Co1-scEDIII-AGA	Upregulated IgG and sIgA response	Bal et al. (2018a)
	Surface display (alive)	Infectious hematopoietic necrosis virus (IHNV)	Glycoprotein (G)	Increased nAb; upregulation of 6 immune-related genes	Zhao et al. (2017)
	Surface display (alive)	*Yersinia enterocolitica* and *Yersinia pseudotuberculosis*	*β1-*integrin binding domain of Invasin	None reported	Kenngott et al. (2016)
	Surface display (alive)	Enterovirus 71 (EV-A71)	pVP1	Upregulated IgG, IgM and IgA response; increased secretion of TNF-α and IFN-γ	Zhang et al. (2016)
	Surface display (dead)	Porcine circovirus Type 2 (PCV2)	PCV2b Cap protein	Upregulated IgG and IgA response	Patterson et al. (2015)
	Surface display (alive)	*Eimeria tenella*	EtMic1 polypeptides	Upregulated IgG response; proliferation of CD4^+^ and CD8^+^ cells	Chen et al. (2015)
	Surface display (alive)	*Eimeria tenella*	EtMic2	Upregulated IgG and sIgA response; proliferation of blood lymphocytes	Sun et al. (2014)
	Surface display (dead)	*Actinobacillus pleuropneumoniae*	ApxI; ApxII	Upregulated IgG and sIgA response	Shin et al. (2013)
	Surface display (alive)	*Candida albicans*	Eno1p	Upregulated IgG response	Shibasaki et al. (2013)
	Subunit (alive and dead)	Dengue virus (DENV)	LTB-scEDIII	Upregulated IgG and sIgA response; proliferation of lymphocytes	Bal et al. (2018b)
	Subunit (dead)	Red-spotted grouper nervous necrosis virus (RGNNV)	Capsid Protein (CP)	Increased nAb	Cho et al. (2017)
	Subunit (alive)	Red-spotted grouper necrosis virus (RGNNV)	Capsid Protein (CP)	Upregulated IgG response	Kim et al. (2014)
*Pichia pastoris*	Surface display (dead)	White spot syndrome virus (WSSV)	PmRab7	None reported	Ananphongmanee et al. (2021)
	Surface display (dead)	White spot syndrome virus (WSSV)	PmRab7 and VP28	None reported	Ananphongmanee et al. (2015)
	Subunit (alive)	Largemouth bass virus (LMBV)	LMBV major capsid protein (MCPD)	Upregulated IgM response	Yao et al. (2022)
	Subunit (alive)	Porcine epidemic diarrhea virus (PEDV)	S1 Region of Spike protein	Upregulated IgG and IgA response	Wang et al. (2016)
	Subunit (alive)	Rock bream iridovirus (RBIV)	Major Capsid Protein (MCP)	Upregulated IgM response	Seo et al. (2013)
	Subunit (dead)	Infectious bursal disease virus (IBDV)	pVP2	Upregulated IgM and IgY response	Taghavian et al. (2013)
*Kluyveromyces lactis*	Subunit (alive)	Porcine reproductive and respiratory syndrome virus (PRRSV)	HP-PRRSV GP5	Upregulated IgG and IgA response; proliferation of lymphocytes	Zhao et al. (2014)

First, *S. cerevisiae* has been used in the fermentation of food and drink for 1,000 of years, with GRAS (“generally regarded as safe”) status in the food industry. Probiotic strains of both *S. cerevisiae* and its conspecific relative, *Saccharomyces boulardii*, have been tested in numerous human clinical trials and have been found to be safe for human consumption (McFarland, 2010; Lam et al., 2019; Pais et al., 2020). Significantly, when they are consumed, these yeasts are known to survive transit through the harsh environment of the gut: When *S. boulardii* was given to healthy volunteers as a probiotic at therapeutic doses (1–2 × 10^10^/d), colonic levels were 2 × 10^8^/g stool (Klein et al., 1993). One possible reason for this is that *S. boulardii* cells, and to a lesser extent, *S. cerevisiae*, are resistant to low pH and bile acid (Fietto et al., 2004; Edwards-Ingram et al., 2007; Cascio et al., 2013; Hudson et al., 2014). As such, these yeasts are prime candidates for a safe and efficacious vaccine delivery system through the harsh environment of the gut.

Second, these yeasts have a relatively long residence time in the human gut: For instance, the probiotic budding yeast, *S. boulardii*, takes 3–5 days to be cleared after oral administration is discontinued (Blehaut et al., 1989; Klein et al., 1993; Elmer et al., 1999). Recall that most food items usually takes just hours to transit the GIT. One paper has reported that orally delivered *S. cerevisiae* are engulfed by dendritic cells, and that the GFP gene packaged in the yeast is released in the mammalian cytosol where it is expressed (Kiflmariam et al., 2013). Another study has shown that recombinant *S. cerevisiae* cells bioencapsulated in *Artemia* could deliver intact antigen to the hindgut of carp larvae and that the antigen could be detected in the mucosal layer of the intestine (Ma et al., 2020). Together, this data suggests that a yeast oral vaccine and its cargo immunogen would have enough time to be absorbed and processed in the gut.

Finally, budding yeast is already known to stimulate the immune system, making it a natural vaccine adjuvant. *Saccharomyces cerevisiae* cells injected subcutaneously induce a robust immunological response in mammals and are avidly taken up by dendritic cells and macrophages *via* phagocytosis (Stubbs et al., 2001; Heintel et al., 2003; Bernstein et al., 2008; Howland et al., 2008; Wansley et al., 2008; Liu et al., 2011). This leads to priming of MHC class I- and class II-restricted, antigen-specific T-cell responses (Stubbs et al., 2001; Bernstein et al., 2008). In contrast, although *S. boulardii* induces a systemic humoral immune response in mice when administered orally, this response is small in magnitude and not directed against *S. boulardii* itself (Hudson et al., 2016). Nonetheless, it is significant that *S. cerevisiae*, administered subcutaneously, and *S. boulardii*, administered orally, have been able to function as adjuvants to enhance the mammalian immune response to vaccines (Grover et al., 2016; Silveira et al., 2017). This suggests that budding yeast may be an ideal oral delivery system, not only because they can efficiently deliver the antigen through the gut and to the immune system, but also because they can inherently stimulate the innate immune system as a stalwart adjuvant as well.

## Emerging yeast oral vaccines for infectious diseases

Currently, there are two basic design strategies for yeast oral vaccine delivery systems that are being deployed against animal and human infectious diseases. The first involves the cell-surface display of peptide or protein antigens, while the second involves the expression and possible secretion of the same.

First, several research teams have used cell-surface display technology to create numerous yeast oral vaccine delivery systems. Cell-surface display directs the expression of target peptides or proteins to the cell surface of a diverse range of cell types through the connection of a protein of interest fused to an anchor protein (Teymennet-Ramírez et al., 2022). Of the 34 papers published in the last 10 years describing a candidate yeast oral vaccine, 26 of them have used cell-surface display to coat the yeast cell with a candidate immunogen (Table 1). These coated yeast cells were then administered orally to animals.

Candidate yeast oral vaccines with cell surface display are being developed against a diverse array of pathogens, including White Spot Syndrome Virus, H5N1 Influenza A Virus, African Swine Fever Virus, and SARS-CoV2, among others, that infect a wide range of animal organisms including shrimp, fish, fowl, swine, and human beings (Table 1). Most of these yeast oral vaccines use the a-agglutinin Aga1p-Aga2p Yeast Surface Display (YSD) system in *S. cerevisiae* (Mei et al., 2017; Zhang C. et al., 2022). Here, the heterologous protein of interest, in our case, the candidate antigen/immunogen, is expressed as a fusion to the Aga2p protein, which *in vivo* is linked to cell-wall covalently-associated Aga1p through two disulfide bonds. A second system uses the alpha-agglutinin Sag1p to anchor the heterologous protein of interest directly to the cell wall (Zhang C. et al., 2022).

The majority of emerging yeast oral vaccines with cell surface display target animal viruses (Table 1). Recently, however, two novel candidate vaccines have been developed against SARS-CoV2 (Gao et al., 2021; Zhang L. et al., 2022). Both candidate COVID-19 vaccines used the receptor binding domain (RBD) of the SARS-CoV2 Spike protein as a candidate immunogen. In one case, the RBD domain was also fused to adjacent the fusion peptide (FP) domain (Zhang L. et al., 2022). Both candidate vaccines were able to elicit robust IgG and IgA responses against the SARS-CoV2 RBD domain. Gao et al. also showed that their candidate vaccine was able to trigger robust cellular immune responses: T-cells proliferated with increased IFN-𝛾 and IL-4 expression. There was also an increase in Th1 bias in memory lymphocytes (Gao et al., 2021). Strikingly, these heightened immune responses were noted even though two distinct vaccination schedules were used suggesting that the precise schedule may not be critical. For one study, mice were orally administered the yeast vaccine on days 1 and 2 for prime immunization and then again on days 14 and 15 for boost immunization (Gao et al., 2021). In contrast, the second SARS-CoV2 study reported that their mice received their oral vaccines on day 1 for prime immunization and then again on days 5, 10, and 21, for boost immunization (Zhang L. et al., 2022). Neither of these studies reported the levels of neutralizing antibodies against SARS-CoV2, and neither vaccine was the subject to a direct challenge animal trial. Both of these will have to be assessed if these vaccine candidates are to proceed to human clinical trials.

Returning now to the other yeast oral vaccines using surface display technology to target non-human animal pathogens, most of the candidate vaccines were able to elicit a strong immune response akin to the responses observed with their SARS-CoV2 counterparts described above. However, many of them were also able to protect vaccinated animals against direct challenge from their target pathogen. These include candidate yeast oral vaccines against red-spotted grouper nervous necrosis virus in convict groupers (Cho et al., 2017); *Helicobacter pylori* in mice (Cen et al., 2021); cyprinid herpesvirus-3 in the common carp (Ma et al., 2020); avian H5N1 influenza virus in chickens (Lei et al., 2021b); and white spot syndrome virus in shrimp (Ananphongmanee et al., 2021), and among others. This growing list of successful animal vaccines suggests that cell-surface display is an effective technology for creating effective yeast oral vaccines.

Next, other research groups are generating subunit yeast oral vaccines where antigenic peptides or proteins are expressed and retained within the budding yeast cell, or are expressed and secreted into the environment. Two emerging yeast oral vaccines of this kind that target animal disease have been recently described.

Recombinant whole budding yeast expressing the capsid protein (CP) of the red-spotted grouper necrosis virus (RGNNV) that were orally administered to mice provoked significantly higher levels of anti-RGNNV IgG antibodies as compared with mice given purified capsid protein (Kim et al., 2014). Moreover, this yeast oral vaccine was able to elicit neutralizing activity against RGNNV while the purified antigen could not. Significantly, when given to fish in a freeze-dried form after disruption, the same CP vaccine was able to reduce mortality in groupers in response to direct challenge with RGNNV (Cho et al., 2017). Neither of these studies included adjuvants to try to enhance the immunogenicity of the antigen. In contrast, another research team fused the *E. coli* heat-labile toxin protein B-subunit (LTB) to the consensus dengue envelope domain III (scEDIII) antigen to create a yeast oral vaccine against dengue virus (Bal et al., 2018b). Both living whole cell (WC) and dead cell-free extracts (CFE) of this oral vaccine were able to stimulate a systemic humoral immune response as well as a mucosal immune response. The team also reported neutralizing activity against DENV-1 and DENV-2, two representative serotypes that cause severe dengue infection, though only the CFE formulation was able to trigger nAbs against the latter serotype. Notably, neither of these subunit vaccines tried to target their antigens directly to the GALT of gut. They simply transported their antigens to the intestines. Nonetheless, there is a report that suggests that the strategy of targeting the candidate antigen to the immune system using peptides that direct the localization of proteins to the GALT is a sound one that could enhance the systemic immune response (Bagherpour et al., 2018).

In sum, though not as common as their cell-surface display counterparts, the few subunit yeast oral vaccines that have been described suggest that they too can elicit the necessary immune response to provide significant protection against challenge by pathogen. It should be interesting to do a head-to-head comparison between two yeast oral vaccines, one surface display and the other a subunit vaccine, that deliver the same antigen to the gut. Which strategy is the more efficacious one?

Finally, a brief comment on whether or not a yeast oral vaccine should be inactivated prior to oral administration. The animal studies described in this review include trials that have tested both living and dead yeast as oral vaccines (Table 1). Moreover, as I noted above, a direct comparison between living WC and dead CFE revealed that both can stimulate the humoral and mucosal immune responses (Bal et al., 2018b), probably because antigens remain intact after inactivation (Arnold et al., 2012). *In toto*, these studies suggest that both living and dead yeast are able to deliver antigen through the stomach in to the gut. In my view, however, one of the advantages of a yeast oral vaccine built upon an FDA approved probiotic is that it can be administered to human subjects alive. The yeast cells that survive transit through the harsh environment of the stomach would be able to continue to grow and to synthesize antigen *de novo* that would trigger the immune system in the small intestine.

## Future directions

In recent years, several candidate yeast oral vaccines have emerged that have provided significant protection against challenge by pathogen. They serve as proof of principle that confirms that this emerging vaccine technology has much promise. In my view, what is most striking about the technology at this time is that there appear to be numerous ways to arrive at an efficacious yeast oral vaccine. Whether you choose to display your antigen or to express it, both strategies work. Whether you use living or dead yeast to orally administer the antigen, both strategies work. Whether you prime and boost just twice or multiple times, both strategies work.

In light of these realities, we should focus now on increasing the efficacy of all of these yeast oral vaccines, regardless of their particular design or immunization schedule. Two specific proposals come to mind. One is to explore the role of adjuvants. Only one published study has linked a known adjuvant—the *E. coli* heat-labile toxin protein B-subunit (LTB)—to a candidate antigen (Bal et al., 2018b). We need to determine if other adjuvants, alone or together, can boost the efficacy of yeast oral vaccines. Another is to determine if known nutritional supplements can increase the viability of the yeast delivery system and thus the efficacy of the vaccine. For example, S-Adenosyl-L-Methionine and trehalose are known to protect budding yeast cells from acid-induced cell death (Malakar et al., 2008; Cascio et al., 2013; Eun Moon et al., 2020). Can they be used as oral supplements to increase efficacy of yeast oral vaccines? Moreover, since the Ras/PKA signal transduction pathway has been implicated in the regulation of yeast cell death in an acidic environment (Lastauskiene et al., 2014), can modulating this pathway enhance yeast oral vaccine efficiency? These questions and others like them can contribute to a research program that can advance these candidate yeast oral vaccines to human clinical trials and beyond.

Finally, there is the question of regulating these yeast oral vaccines as genetically modified organisms (GMO). Legislation regulating GMO throughout the world is complex and diverse. However, there have been recent calls to simplify them so that advanced therapy medicinal products (ATMPs), such as gene therapies and vaccines that consist of or contain GMOs, can be brought to clinical trials (Kauffmann et al., 2019; Beattie, 2021).

## Author contributions

The author confirms being the sole contributor of this work and has approved it for publication.

## Funding

NA received a grant from the Department of Science and Technology of the Republic of the Philippines. However, the grant does not include any funds for payment of open access fees.

## Conflict of interest

The author declares that the research was conducted in the absence of any commercial or financial relationships that could be construed as a potential conflict of interest.

## Publisher’s note

All claims expressed in this article are solely those of the authors and do not necessarily represent those of their affiliated organizations, or those of the publisher, the editors and the reviewers. Any product that may be evaluated in this article, or claim that may be made by its manufacturer, is not guaranteed or endorsed by the publisher.
